# Prevention of Cervical Cancer in Sub-Saharan Africa: The Advantages and Challenges of HPV Vaccination

**DOI:** 10.3390/vaccines6030061

**Published:** 2018-09-08

**Authors:** Eleanor Black, Robyn Richmond

**Affiliations:** School of Public Health and Community Medicine, University of New South Wales, Sydney 2052, Australia; R.Richmond@unsw.edu.au

**Keywords:** cervical cancer, HPV vaccination, sub-Saharan Africa

## Abstract

Cervical cancer is a critical public health issue in sub-Saharan Africa (SSA), where it is the second leading cause of cancer among women and the leading cause of female cancer deaths. Incidence and mortality rates are substantially higher than in high-income countries with population-based screening programs, yet implementing screening programs in SSA has so far proven to be challenging due to financial, logistical, and sociocultural factors. Human Papillomavirus (HPV) vaccination is an effective approach for primary prevention of cervical cancer and presents an opportunity to reduce the burden from cervical cancer in SSA. With a number of SSA countries now eligible for Global Alliance for Vaccines and Immunization (GAVI) support for vaccine introduction, it is timely to consider the factors that impede and facilitate implementation of vaccine programs in SSA. This article describes epidemiological features of cervical cancer in SSA and the current status of HPV vaccine implementation in SSA countries. Rwanda’s experience of achieving high vaccination coverage in their national HPV immunization program is used as a case study to explore effective approaches to the design and implementation of HPV vaccination programs in SSA. Key factors in Rwanda’s successful implementation included government ownership and support for the program, school-based delivery, social mobilization, and strategies for reaching out-of-school girls. These findings might usefully be applied to other SSA countries planning for HPV vaccination.

## 1. Introduction

Cervical cancer is a major cause of morbidity and mortality in sub-Saharan Africa (SSA), where the burden from noncommunicable diseases is increasing due in large part to improved control of communicable diseases and an ageing population [1]. Incidence and mortality rates for cervical cancer are extremely high in SSA [2], reflecting disparities in the effectiveness of prevention and control strategies in high-income versus low- and middle-income countries (LMICs). While high-income countries have been able to implement and sustain population-based screening programs with high population coverage, screening has been less effective in SSA due to financial, logistical, and sociocultural barriers [3]. Additional preventive strategies are needed to address the growing burden from cervical cancer. The introduction of the Human Papillomavirus (HPV) vaccine has allowed for effective primary prevention of cervical cancer and has already demonstrated important impacts on reducing incidence in high-income countries [3,4]. HPV vaccination is a promising preventive measure for SSA, and a number of SSA countries have implemented or are planning for the implementation of HPV vaccination programs [5,6,7]. In this article, we describe the significant burden from cervical cancer in SSA and the role for HPV vaccination. We summarize the current status of pilot and national HPV vaccination programs in SSA countries, and the challenges to successful implementation. Factors that have facilitated some countries, such as Rwanda, to overcome these challenges and achieve high vaccine coverage are explored. This article aims to provide a current comprehensive summary of the advantages and challenges of HPV vaccination in SSA in order to provide guidance for those involved in the development and implementation of HPV vaccination programs in SSA.

Cervical cancer is the second most frequent cause of female cancer in women aged 15–44 years in SSA [2]. The most recent data from the International Agency for Research on Cancer (IARC) indicates that there were 93,225 new cases in 2012 (compared with 94,378 cases of breast cancer, the most frequent female cancer in SSA) [2,8]. There is considerable geographic variation however, and cervical cancer is more common than breast cancer in several SSA countries, as well as in the Eastern and Central regions of SSA [2,8] (see Appendix A for regional classification of countries). Malawi, Mozambique, Comoros, Zambia, Zimbabwe, and Tanzania have among the highest age-standardized incidence rates in the world at over 50 cases per 100,000 women annually [3,8]. Age-standardized incidence rates in high-income countries are much lower, for example 5.5 per 100,000 in Australia and 6.6 per 100,000 in the United States of America [2]. Cervical cancer is the leading cause of female cancer deaths overall in SSA, with 57,381 deaths reported in 2012 [2]. Age-standardized incidence and mortality rates are highest in Eastern Africa, which also has the highest HPV prevalence in the general population (20.5%, compared with 18.6% in SSA overall and a global prevalence of 4.1%) [8] (see Table 1).

Country-specific data on time trends are limited but where available demonstrate an increasing burden from cervical cancer. In Uganda, cervical cancer prevalence has increased by 1.8% per annum over the previous two decades to 2018 [9], and the annual crude incidence rate in Uganda increased from 80 cases per 100,000 women in 1993 to over 180 cases per 100,000 women in 2007 among women aged 45 and over [8]. In all regions of SSA, age-standardized incidence rates increase between the ages of 40 to 44 and peak between the ages of 55 to 65 at just over 100 women per 100,000. This is in contrast to high-income countries where the peak incidence is lower, at around 20 women per 100,000, and is observed at a younger age (40–44) [3]. These differences can be explained by the effect of cervical cancer screening and HPV vaccination programs, which in high-income countries have successfully led to improved control of the disease [6,10].

Cervical cancer is usually preceded by a long phase of precancerous changes, which can be detected by screening before progression to invasive disease, allowing for early intervention and treatment [11]. In high-income countries, screening by cytology (or ‘Pap smears’) has been the cornerstone of cervical cancer prevention since the 1940s [12]. National screening programs using cytology have resulted in prevention of up to 80% of cervical cancers in high-resource settings [10,12], but screening programs have been less effective at controlling cervical cancer in SSA due to financial, logistical, and sociocultural constraints to implementation [13]. Many countries in SSA do not have screening programs, and among those that do, coverage rates are frequently low [13,14]. In Uganda, lifetime screening rate estimations for cervical cancer are between 4.8% to 30% [14,15]. Mechanisms for quality assurance and invitations to screen are lacking in most of SSA [8].

Screening methods used vary between countries in SSA. The most frequent method used is Pap smears with cytological analysis [8], but SSA faces challenges in achieving high screening coverage with this method due to inadequate infrastructure and lack of trained personnel [1,15]. Alternative screening methods include visual inspection of the cervix with acetic acid (VIA) or Lugol’s iodine (VILI), as well as HPV DNA testing [11]. These are well recognized as viable alternatives to Pap smears in low-resource settings [8,10], and ‘screen-and-treat’ approaches using either HPV testing or VIA followed by cryotherapy have been shown to be a cost-effective prevention strategy [16]. However, the sensitivity of visual inspection methods is provider-dependent and highly variable, and VIA and VILI are frequently inaccurate in women over 50 years of age due to changes in the cervical epithelium [1]. While HPV testing has been shown in numerous studies to be extremely sensitive, it has high infrastructure requirements and is not yet widely available in SSA [1]. 

The high prevalence of HPV infection in SSA points to a great need for improving access to effective cervical cancer screening. While alternative screening types suitable for LMICs now exist, challenges to implementation have meant they are still not widely available and uptake of screening remains low [14]. Primary prevention of cervical cancer with HPV vaccination is thus particularly important in SSA [17]. 

## 2. Advantages of HPV Vaccination for SSA

Infection with the Human Papillomavirus (HPV) is the main cause of cervical cancer, with some estimates that it is responsible for greater than 99% of cases [10], and the development of prophylactic HPV vaccines has made primary prevention of cervical cancer possible [1,6,10]. The first national HPV vaccination programs began in high-income countries in 2007, and vaccines have now been licensed in over 100 countries [8]. HPV vaccines have been shown to be at least 95% effective in preventing infection with the most oncogenic strains of HPV, and 100% effective in preventing precancerous changes of the cervix when administered prior to the onset of sexual activity [1,5]. In countries with national immunization programs, girls aged nine to twelve are generally targeted [6]. Since 2014, the World Health Organization (WHO) has recommended a two-dose schedule instead of a three-dose schedule, which reduces delivery costs while maintaining high immunogenic protection [5,6].

HPV vaccination holds great potential for reducing the burden from cervical cancer in LMICs. It has been demonstrated to be cost-effective, for example Bray and colleagues (2015) estimated that the cost of averting one disability-adjusted life-year (DALY) with HPV vaccination is less than the per capita gross domestic product (GDP) [18]. There is already robust evidence that HPV vaccination reduces cervical cancer incidence, and a recent systematic review and meta-analysis demonstrated that HPV prevalence decreased significantly when countries achieved coverage rates of over 50% [19]. Reports from the Program for Appropriate Technology in Health (PATH) HPV vaccine demonstration projects indicate that the vaccine is acceptable in LMICs [1]. 

Over the past decade, HPV vaccination efforts have gained momentum in SSA, in large part due to inclusion of the HPV vaccine in the GAVI portfolio [4,5,19,20]. GAVI-eligible countries can apply for HPV vaccines and funding assistance for the HPV vaccination demonstration project, and most countries in SSA are eligible for GAVI support [20,21]. Kenya, Zimbabwe, Benin, Burundi, Cameroon, Rwanda, and Uganda are some of the countries that have been supported by GAVI to implement demonstration or national programs [8,21]. 

As of June 2018, there are eight countries in SSA with a national HPV immunization program (see Table 2), and nearly twice as many with pilot programs [8,22]. Data on vaccine coverage in SSA countries with pilot or national programs is limited, and not available for all countries [6,20] (see Table 2). Rwanda achieved extremely high vaccine coverage of greater than 98% for the 3-dose schedule of vaccinations in 2014 [8,23]. Table 2 lists the SSA countries with national HPV immunization programs, their delivery platforms, and the most recent estimated coverage where available.

## 3. Challenges to HPV Vaccination in SSA

Numerous barriers to implementation of national vaccination programs in SSA exist, including inadequate infrastructure and finances, limited health worker training, vaccine cost, and cold chain capacity constraints [1,24]. As young girls are the target group for the vaccine, an appropriate delivery platform is required, and further barriers include sociocultural issues such as the stigmas associated with HPV being a sexually transmitted infection [22]. Lack of knowledge of HPV vaccination and associated misconceptions also make implementation challenging. In a cross-sectional, mixed-methods study of factors influencing HPV uptake in Eastern Uganda, Nabirye and colleagues found that lack of awareness about the vaccine was the main reason given by adolescent girls for not being vaccinated [9]. Misconception of the HPV vaccine and fear of pain and adverse events have been reported as barriers for adolescents and parents in Tanzania, Botswana, and Mozambique [22,25].

Rwanda was the first low-income country to implement a national HPV vaccination program [24,26]. Rwanda’s national cervical cancer prevention program, launched in 2011, includes HPV vaccination for girls aged 12 to 15 [7]. As demonstrated in Table 2, Rwanda has achieved extremely high coverage rates, which surpass those of many high-income countries [8]. Rwanda’s experience implementing its national HPV vaccination program illustrates how some of the challenges can be overcome in SSA and other low-resource settings. 

The Rwandan Ministry of Health (MOH) received support from *Merck*, an HPV vaccine manufacturer. *Merck* provided free vaccines to Rwanda for three years, with an understanding that future doses would be supplied at a concessional fee [26]. Rwanda’s national HPV vaccination program was planned with extensive input from a variety of stakeholders including the Ministry of Education (MOE) and health workers. A nationwide sensitization campaign was rolled out before vaccinations began [7,23] (see Table 3), and the program was implemented without a prior demonstration project [22]. Schools were used as the delivery platform, which was effective as school attendance in Rwanda is greater than 98% [7]. The target group was identified by grade rather than age, as many girls were unsure of their age [23]. Community health workers identified and vaccinated girls not attending school, through registration records from health centres [26]. 

The strategies used by Rwanda have been utilized successfully in other LMICs introducing national HPV vaccination programs. Gallagher and colleagues [27] undertook an ecological study of 45 LMICs with HPV demonstration projects or national programs to understand what factors were related to successful experiences and high vaccine coverage. They found that countries that had achieved high coverage had delivered the program through MOH ownership, and often with partner support, had involved the MOE, and had used schools as the delivery platform for vaccination. They had also included a strategy to reach out-of-school girls and employed good social mobilization and community engagement strategies [27]. Ladner and colleagues analyzed data from the Gardasil Access Program in 14 LMICs and found that higher vaccine uptake rates were significantly associated with programs that involved community follow-up of vaccinated girls, and programs that were based in schools [28].

## 4. Strategies for Successful HPV Vaccine Implementation in SSA

With eight SSA countries having now introduced national HPV vaccination programs, and at least twice that number with current pilot projects, it is important to learn from countries like Rwanda that have had successful experiences with implementation. In order to achieve high vaccine coverage sustained over time, the following lessons from Rwanda’s experience could be useful for other SSA countries. 

### 4.1. Government Ownership and Support

High levels of commitment at the national governmental level with demonstrated leadership are important for successful HPV introduction [22]. A recent collaborative project involving 17 SSA countries identified factors that facilitated successful HPV vaccination implementation. Many of the participant countries reported that government support was critical for successful HPV introduction [22]. Government commitment and leadership was strengthened and exemplified through patronage of the first Ladies in a number of countries including South Africa, Mozambique, Tanzania, and Rwanda. Participant countries also reported that financial and logistical support from international partners such as GAVI was extremely important for implementation [22]. 

### 4.2. School-Based Delivery with MOE Involvement

Delivery through schools is effective in countries with high levels of school attendance [9,29]. In HPV vaccination demonstration projects in LMICs, final dose coverage levels have been higher in countries delivering vaccines through schools compared with health facilities [6]. Uganda’s HPV demonstration project explored two possible strategies for HPV vaccine delivery: offering the vaccine through healthcare facilities, and offering it at schools. For its national program, Uganda used the school-based platform for delivery, as this was found to be more effective [22]. School-based delivery has the advantages of easy identification and access to the target population [22], although effective strategies for identifying out-of-school girls must also be in place. Using schools as a delivery platform requires collaboration between the MOH and the MOE, a key feature of Rwanda’s implementation process. 

### 4.3. Strategies for Reaching Out-of-School Girls

High levels of school attendance facilitated Rwanda’s success with school-based delivery, and community health workers were able to identify and vaccinate out-of-school girls at health centres. In countries with lower school attendance, reaching out-of-school girls may be more challenging, and many SSA countries have indicated difficulties in achieving this [22]. There is a need for research into how to reach out-of-school girls in these countries, as the effectiveness of the HPV vaccine depends on high coverage.

### 4.4. Social Mobilisation 

Early community involvement and social mobilization was a cornerstone of Rwanda’s HPV vaccination program [7,26]. A sensitization campaign was delivered prior to vaccine introduction and there was early distribution of information and education through community leaders, teachers, and health workers [24]. Dissemination of knowledge is critical to successful implementation of vaccination programs. A recent cross-sectional study of adolescents and health workers from Uganda found that vaccine uptake in the study population was only 14%, and both health workers and adolescents had limited awareness of the importance of the vaccination [9]. 

## 5. Conclusions

The burden of cervical cancer in SSA is substantial, and there is a great need for improved prevention in order to reduce morbidity and mortality from this disease. While secondary prevention through national screening programs has contributed significantly to reducing incidence and mortality from cervical cancer in high-income countries, constraints to implementing and sustaining similar programs in SSA have limited the effectiveness of screening in SSA. Primary prevention of cervical cancer through HPV vaccination is a cost-effective preventive measure that is currently being implemented in numerous SSA countries. Although challenges exist, examples of high vaccine coverage and sustainable programs demonstrate that high vaccine coverage and sustainable programs can be delivered with strong commitment from government, comprehensive planning with ministries of health as well as education, and early community sensitisation. These lessons might usefully be applied in SSA countries with current HPV vaccine demonstration projects, as well as those beginning to plan for vaccine introduction.

## Figures and Tables

**Table 1:** Key Epidemiological Data for Cervical Cancer and HPV for SSA regions [2,8].

Burden of cervical cancer and HPV infection	SSA	Eastern Africa	Central Africa	Western Africa	Southern Africa
Annual number of new cervical cancer cases	93,225	45,707	11,540	8652	8652
Age-standardized incidence rate per 100,000 women	34.8	42.7	30.6	31.5	31.5
Annual number of cervical cancer deaths	57,381	28,197	7917	4721	4721
Age-standardized mortality rate from cervical cancer per 100,000 women	22.5	27.6	22.2	17.9	17.9
HPV prevalence (%) in the general population	18.6	20.5	9.8	17.9	17.9

**Table 2:** National HPV Immunization Programs in SSA [8,9].

Country	Year of Introduction	Delivery Platform	Estimated Coverage
Botswana	2015	School-based (grades 5–7) and out-of-school girls aged 9–13	-
Lesotho	2012	School-based	-
Mauritius	2016	School-based (grade 5)	-
Rwanda	2011	School-based (grade 6) and out-of-school girls	HPV3 98.7% (2014)
Senegal	2016	School-based	-
Seychelles	2014	School-based (grade 6)	HPV1 77% HPV2 76% (2014)
South Africa	2014	School-based (grade 4)	HPV1 92% HPV2 72% (2014)
Uganda	2015	School-based (grade 4) and out-of-school girls aged 10	-

HPV1 = 1 dose of vaccine; HPV2 = 2 doses; HPV3 = 3 doses.

**Table 3:** Key Features Contributing to the Success of Rwanda’s HPV Vaccination Program [7,23,26].

Feature	Key Examples
Preparation	Multidisciplinary involvement in development of program plan, e.g., Ministry of Education and Centre for Epidemiology. Planning involved identifying financial requirements; identification of target population; health worker training; and information dissemination strategies Ministry of Education involved in developing and implementing school-based vaccination
Communication and Social Mobilisation	Sensitisation campaign delivered prior to vaccine introduction: information provided by health workers, government, and media Teachers and health workers informed students about the vaccine Community partnership with inclusion of local leaders, community health workers and teachers
Delivery	Schools used as delivery platform targeting girls by grade rather than age High school enrolment (>98%) facilitated high vaccine coverage Multiple phases to vaccination, following girls over time and providing extra vaccination opportunities for girls who had missed the initial phases
Sustainability	Phased national implementation rather than demonstration projects made feasible through agreement with Merck Three years of cost-free vaccines provided by Merck, and future vaccine doses provided at concessional prices; consequently less uncertainty over availability of future financing

## Appendix A

**Table A1:** Regions of Africa.

Eastern Africa	Central Africa	Western Africa	Southern Africa
Burundi Comoros Djibouti Eritrea Ethiopia Kenya Madagascar Malawi Mauritius Mozambique Rwanda Seychelles Somalia South Sudan Tanzania Uganda Zambia Zimbabwe	Angola Cameroon Chad Congo DR Congo Equatorial Guinea Gabon S. Tome & Prin.	Benin Burkina Faso Cote d’Ivoire Gambia Ghana Guinea Guinea-Bissau Liberia Mali Mauritania Niger Nigeria Senegal Sierra Leone	Botswana Lesotho Namibia South Africa Swaziland
